# Chinese Herbal Medicine Image Recognition and Retrieval by Convolutional Neural Network

**DOI:** 10.1371/journal.pone.0156327

**Published:** 2016-06-03

**Authors:** Xin Sun, Huinan Qian

**Affiliations:** Beijing University of Chinese Medicine, Academy of Basic Medicine Sciences, Beijing, China; Jiangnan University, CHINA

## Abstract

Chinese herbal medicine image recognition and retrieval have great potential of practical applications. Several previous studies have focused on the recognition with hand-crafted image features, but there are two limitations in them. Firstly, most of these hand-crafted features are low-level image representation, which is easily affected by noise and background. Secondly, the medicine images are very clean without any backgrounds, which makes it difficult to use in practical applications. Therefore, designing high-level image representation for recognition and retrieval in real world medicine images is facing a great challenge. Inspired by the recent progress of deep learning in computer vision, we realize that deep learning methods may provide robust medicine image representation. In this paper, we propose to use the Convolutional Neural Network (CNN) for Chinese herbal medicine image recognition and retrieval. For the recognition problem, we use the softmax loss to optimize the recognition network; then for the retrieval problem, we fine-tune the recognition network by adding a triplet loss to search for the most similar medicine images. To evaluate our method, we construct a public database of herbal medicine images with cluttered backgrounds, which has in total 5523 images with 95 popular Chinese medicine categories. Experimental results show that our method can achieve the average recognition precision of 71% and the average retrieval precision of 53% over all the 95 medicine categories, which are quite promising given the fact that the real world images have multiple pieces of occluded herbal and cluttered backgrounds. Besides, our proposed method achieves the state-of-the-art performance by improving previous studies with a large margin.

## Introduction

Chinese herbal medicine image recognition and retrieval have great potential of applications. Given a herbal medicine image, herbal medicine image recognition aims to recognize its medicine category, while herbal medicine image retrieval aims to find its similar images. In the traditional way, recognizing medicine category based on herbal medicine images requires professional knowledge, and the hundreds of medicine categories make it difficult for beginners. Besides, when collecting some medicine images, retrieving herbal medicine images with the text-only way is not reliable because sometimes the names of medicine are not even known. Here, we come up with a question: why not make life easier by recognizing and retrieving herbal medicine images automatically? The images contain all the information we need, while the problem is how to understand them with machines. Therefore, recognizing and retrieving medicine images has become an urgent need. In this paper, we focus on these two topics.

Previous work on herbal medicine image retrieval is little, while researchers have done some studies on the recognition problem, and most of them use hand-crafted low-level image features [1–4]. Li [5] proposed to use different low-level features for medicine recognition, such as shape, color and texture features, and they combined all these low-level features to improve recognition performance. Tao *et al*. [6–11] considered texture as a key factor, and they designed various texture features from different aspects for herbal medicine recognition. However, there are two main limitations in these studies. Firstly, they only consider images with a single clean herbal without any background. This setting is too ideal since real world herbal images usually have multiple pieces of mutually occluded herbal and complex backgrounds, which make these studies difficult to use in practical applications. Secondly, due to the cluttered backgrounds, the above low-level features cannot be reliable as they are easily changed with the varying backgrounds. Therefore, designing a robust image representation for real world herbal medicine image recognition and retrieval is in desperate need.

In the computer vision community, how to design a robust image representation has always been a hot topic in the past decade [12–31]. Researchers first used low-level features, such as gradient [12, 32], color [17], shape [14] and texture [13], but these features are easily affected by image appearance. To improve the robustness, the Bag-of-Words (BoW) model was proposed to construct mid-level image representation [18–25], but it cannot recognize a large number of categories. In recent years, with the development of parallel computing, the Convolutional Neural Network (CNN) that was proposed several decades ago has re-dominated the area of image recognition [26–31, 33–35]. Due to the deep structure, CNN can construct high-level image representation, which is similar to the semantic understanding of humans, thus it is able to distinguish many categories and robust to background influence. Inspired by the robust image representation of CNN, we believe CNN is appropriate for herbal medicine recognition and retrieval, especially for the real world herbal medicine images with cluttered backgrounds and a large number of medicine categories.

**Fig 1:**
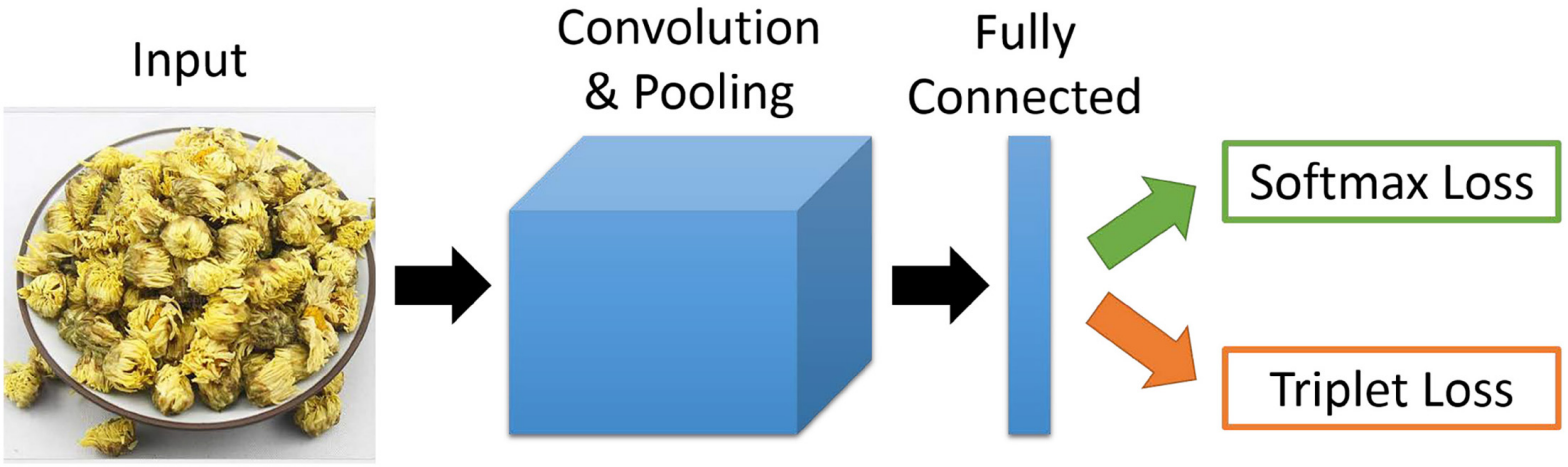
The framework of the Convolutional Neural Network (CNN) for Chinese herbal medicine image recognition and retrieval. CNN model mainly consists of convolutional, pooling, fully-connected and loss layers. For recognition, we train the CNN model with the softmax loss; while for retrieval, we fine-tune the recognition work by adding a triplet loss.

In this paper, we propose to use the Convolutional Neural Network (CNN) for herbal medicine image recognition and retrieval. Especially, we construct a public database of real world Chinese herbal medicine images for use in research and practical applications. In our database, we collect herbal medicine images from the Internet with cluttered backgrounds, and 95 herbal medicine categories with 5523 images are included. The whole training procedure is shown in Fig 1. For the medicine recognition process, we train a VGG16-like [29] recognition network with the softmax loss, which optimizes that each image should be given the correct category. Then, in medicine retrieval, we fine-tune the recognition network with the triplet loss, which guarantees that the images of the same category and similar appearance can be found firstly.

In the experimental evaluation, we split the database into training and test set, in which 50%∼90% is for training and the rest for test. Experimental results show that our method can achieve the average recognition precision of 71% and the average retrieval precision of 53% on all the medicine categories, and given the fact that most images have multiple pieces of mutually occluded herbal and cluttered backgrounds, the above results are quite promising and show the robust image representation of CNN in Chinese medicine. Besides, our proposed method achieves the state-of-the-art performance by improving previous studies with a large margin.

This paper has two main contributions:

- Compared to the previous studies which only consider images with a single clean herbal without backgrounds, we construct a public database of herbal medicine images with multiple pieces of mutually occluded herbal and cluttered backgrounds, which makes our method appropriate for practical applications.
- Compared to the recognition ability of previous studies, we use CNN to largely boost the recognition precision in real world medicine images, which has the potential for applications. Besides, we get promising results for herbal medicine image retrieval, which was rarely considered in the literature of Chinese medicine.

The rest of this paper is organized as follows. Sec.2 will introduce some related work on Chinese herbal medicine image recognition and retrieval. Then, in Sec.3, the construction of the database and the whole method will be elaborated. To evaluate the proposed method, Sec.4 will give detailed experimental results. Finally, we summarize the paper with conclusive remarks in Sec.5.

## Related Work

In this section, we will give a detailed review of the studies on Chinese herbal medicine image recognition and retrieval. Overall speaking, there is little study on herbal image retrieval, while there are some on herbal image recognition. Therefore, we mainly introduce the previous work on herbal image recognition, and simply review image retrieval in the computer vision community.

Previous studies on Chinese herbal medicine image recognition mainly use hand-crafted low-level image features [1–11]. Li [5] proposed to use different low-level features for medicine recognition, such as shape, color and texture features. They trained a classifier for each kind of feature, and combined these features by weighting the confidence of each classifier to predict the medicine category. However, they only considered 5 herbal medicine categories, which is too little for research and applications. As the herbal medicine category becomes more, Tao *et al*. [6–11] found that color and shape features are not reliable because many herbal medicine categories have the similar color and shape, while different medicine categories have different textures. Therefore, they proposed to use various texture features from different aspects to describe the herbal images, and obtained promising recognition precision on 18 herbal medicine categories.

However, there are two main limitations in the above studies. Firstly, they only consider clean herbal medicine images without any background, and all images only contain one piece of herbal at the center. This experimental condition is too ideal because in real world situations, complex backgrounds and multiple pieces of mutually occluded herbal will be included, which makes previous studies very difficult to use in practical applications. Secondly, due to the cluttered backgrounds, the above low-level features cannot be reliable as they are easily changed. Gradient, shape, color and texture are all extracted directly from image pixels without high-level semantic information, thus these features will not remain stable as the pixels in backgrounds change dramatically.

Recently, due to the great progress of deep learning methods, the development of image recognition has entered a new stage. Among the deep learning models, the Convolutional Neural Network (CNN) has largely boost the performance of image recognition, especially in the computer vision community [26–31, 36, 37]. Due to the deep structure of the network, CNN can extract high-level image representation, which is able to distinguish many medicine categories and robust to background influence. Therefore, inspired by the great discrimination of CNN, we attempt to use CNN in Chinese herbal medicine image recognition and retrieval. At the same time, we construct a public database of real world herbal medicine images to valid our method, and also for research and applications.

For the retrieval problem, due to the little study in the area of Chinese medicine, we simply review some work in computer vision. To train a retrieval model, the simplest way is to use a recognition model, which considers the image with the same category as the similar images [30]. However, belonging to the same category is not enough because these images may have very different image appearance, *e.g.*, the same dog category may have very different appearance. Therefore, it is necessary to define how similar between images, which gives the motivation of using order information in training the retrieval model. Among the methods using order information, the typical method is using the triplet loss, which is widely used in the retrieval task [38–46]. Combined with the great discrimination of CNN, the retrieval model will find images that are both semantic similar and appearance similar. Therefore, we believe that combining CNN with the triplet loss is appropriate for Chinese herbal medicine image retrieval.

## Methods

In this section, we will introduce our main method. We first introduce how to construct the database, then we will elaborate the method in detail.

### Database Construction

A good database is important for developing good methods and leading the methods to practical applications. In the area of Chinese herbal medicine recognition, no such database is public to researchers, thus it is hard to evaluate different methods with the same standard. Besides, previous studies only consider images with a single clean herbal without any background, which is a too ideal setting and makes them difficult to use in applications. Therefore, we propose to construct a public database of Chinese herbal medicine images for research and applications.

**Fig 2:**
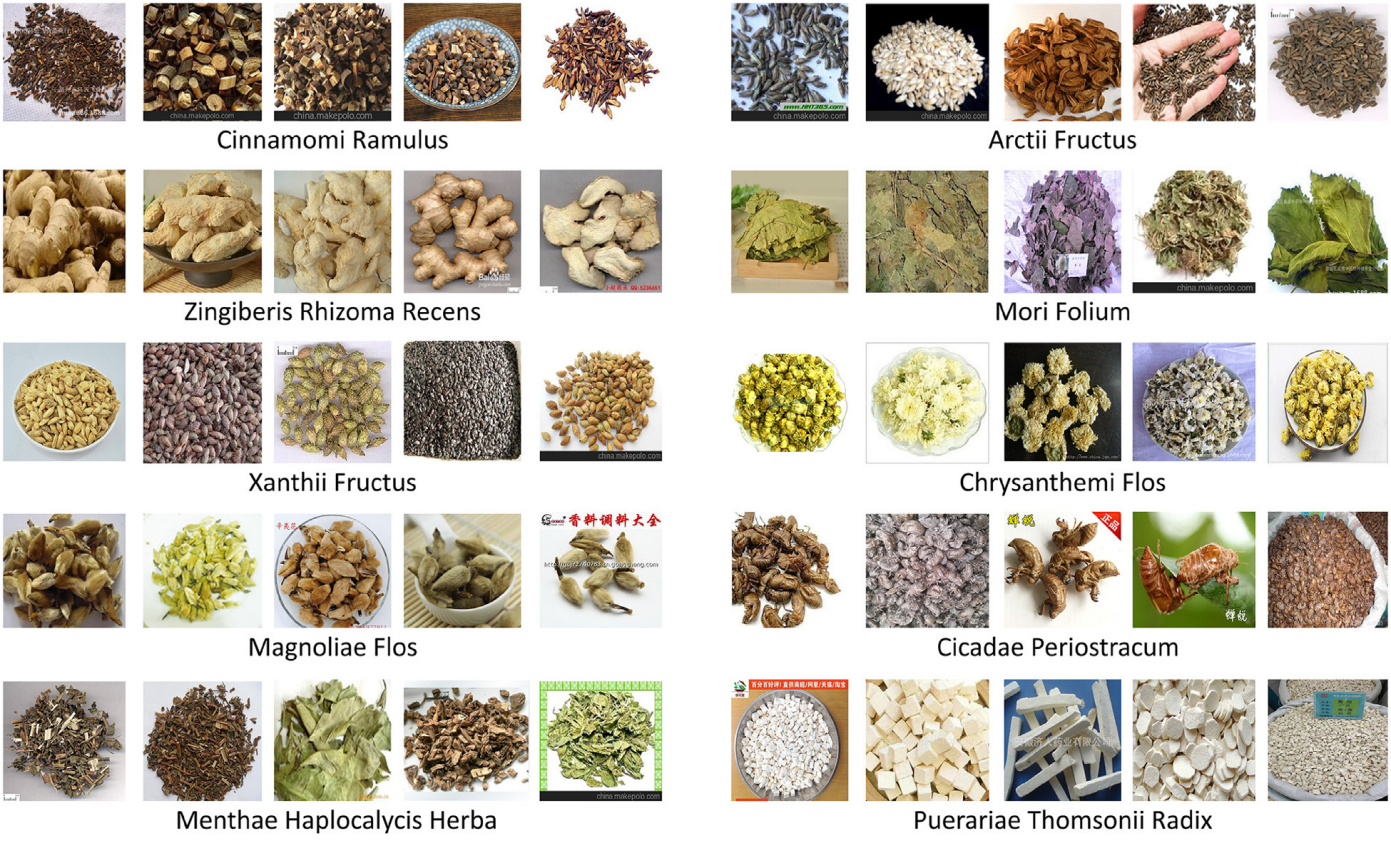
Some herbal medicine images in our database. We list 10 medicine categories, whose corresponding Latin names are given below.

To construct the database, we select 100 common Chinese medicine categories, and use their names as keywords to search for medicine images in Google. For each category, we select the top 100 ranking images as candidates because most of the low ranking images are noisy. Then, we check the candidates carefully to preserve the effective ones, whose number varies from 30 to 90 for each category. Finally, we get 5523 herbal medicine images with 95 categories in total. Fig 2 shows some images in our database, and we can see clearly that most of these images have multiple pieces of mutually occluded herbal and various backgrounds, which are more appropriate for research and applications. The database will be available online soon.

### Image Recognition

In this part, we elaborate how to use CNN for Chinese herbal medicine image recognition. CNN is a neural network that consists of different layers, and four layers are commonly used: convolutional layers, pooling layers, fully-connected layers and loss layers. (1) Convolutional layer is made of filters, which are applied on a whole image to capture local information. (2) Pooling layer aims to down-sample the image to make it smaller, and it is usually located after the convolutional layer. (3) Fully-connected layer aims to integrate the local semantic information of the convolutional layers into global semantic information. It is usually located after pooling layers to further reduce the dimensionality of image representation. (4) Loss layer is the optimization objective which instructs the learning process, in which the softmax loss is commonly used for the recognition problem.

Due to the large amount of parameters in CNN, training it usually requires a large number of labeled images. However, we only label 5523 images in our database, thus training CNN directly will not work. Alternatively, one strategy for this problem is called fine-tuning, which uses the pre-trained CNN as initialization and learns a new CNN model based on it. In this way, not many labeled data is required and the pre-trained CNN can be adapted easily to our task. Here, we use the CNN model called VGG16-net, which is pre-trained on the ImageNet 2012 dataset containing more than 1.2 million Internet images with 1000 object categories. Due to the very deep structure, VGG16-net has obtained very promising recognition precision, thus we use the VGG16-net as the pre-trained model for our recognition network.

Table 1 gives the structure of the VGG16-net, in which there are six main stages and in total 16 weight layers (*conv* + *FC*). *conv* means the convolutional layer, *max*–*pool* denotes the max pooling layer and *FC* is the fully-connected layer. Besides, *conv*3–64 means that this convolutional layer has the filter size of 3 ∗ 3 with 64 channels, and *FC*−4096 denotes that this fully-connected layer has 4096 neurons.

**Table 1:** The structure of the VGG16-net, which has six main stages. conv means convolutional layer, max-pool denotes max pooling layer and FC is the fully-connected layer.

1	conv3-64	conv3-64	max-pool	
2	conv3-128	conv3-128	max-pool	
3	conv3-256	conv3-256	conv3-256	max-pool
4	conv3-512	conv3-512	conv3-512	max-pool
5	conv3-512	conv3-512	conv3-512	max-pool
6	FC-4096	FC-4096	FC-1000	

**Fig 3:**
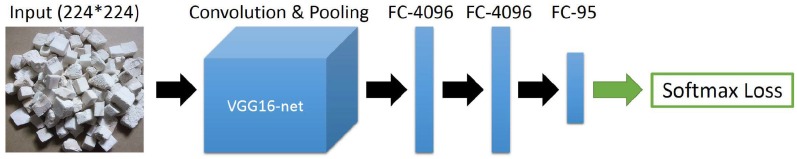
An illustration of training the recognition CNN model based on the VGG16 model pre-trained on ImageNet 2012 dataset.

Fig 3 shows how we train the recognition CNN model with the softmax loss. In the last fully-connected layer, we change the number of neurons to be 95, which equals the number of medicine categories in our database, and each represents the possibility of the image belonging to a medicine category. The database contains variable resolution images, but our network requires a constant input dimensionality. Therefore, we down-sample the images to a fixed resolution of 224 ∗ 224, which is the input image size of the VGG16-net. Given a rectangular image, we first re-scale the image to 256 ∗ 256, in which the 224 ∗ 224 patch is then cropped out as the input image. In this way, we can largely increase the number of training images, which is better to train the CNN model.

### Image Retrieval

**Fig 4:**
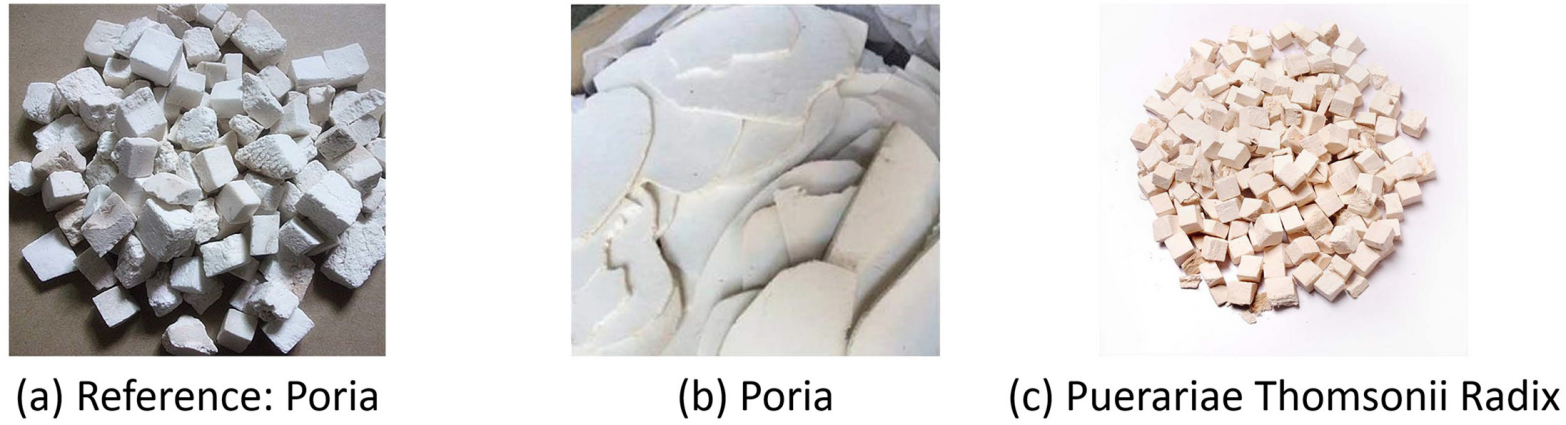
An illustration of the semantic similar and appearance similar. The reference image in (a) is the Poria, the image in (b) is the Poria, and the image in (c) is the Puerariae Thomsonii Radix. Although (b) and (a) share the same medicine category, they look very different; (c) and (a) belong to different categories, but they look very similar.

In this part, we explain how to use CNN for Chinese herbal medicine image retrieval. As mentioned above, only image recognition cannot guarantee an accurate search, because the images with the same medicine category may have very different appearance, and the images with different medicine categories may have very similar appearance. Fig 4 shows an example, in which Fig 4(b) has the same category with Fig 4(a), but they look very different; Fig 4(c) and 4(a) have different categories, but they look very similar, thus it is difficult to distinguish these samples with only the category information, and necessary to consider both semantic and appearance information in the retrieval process.

**Fig 5:**
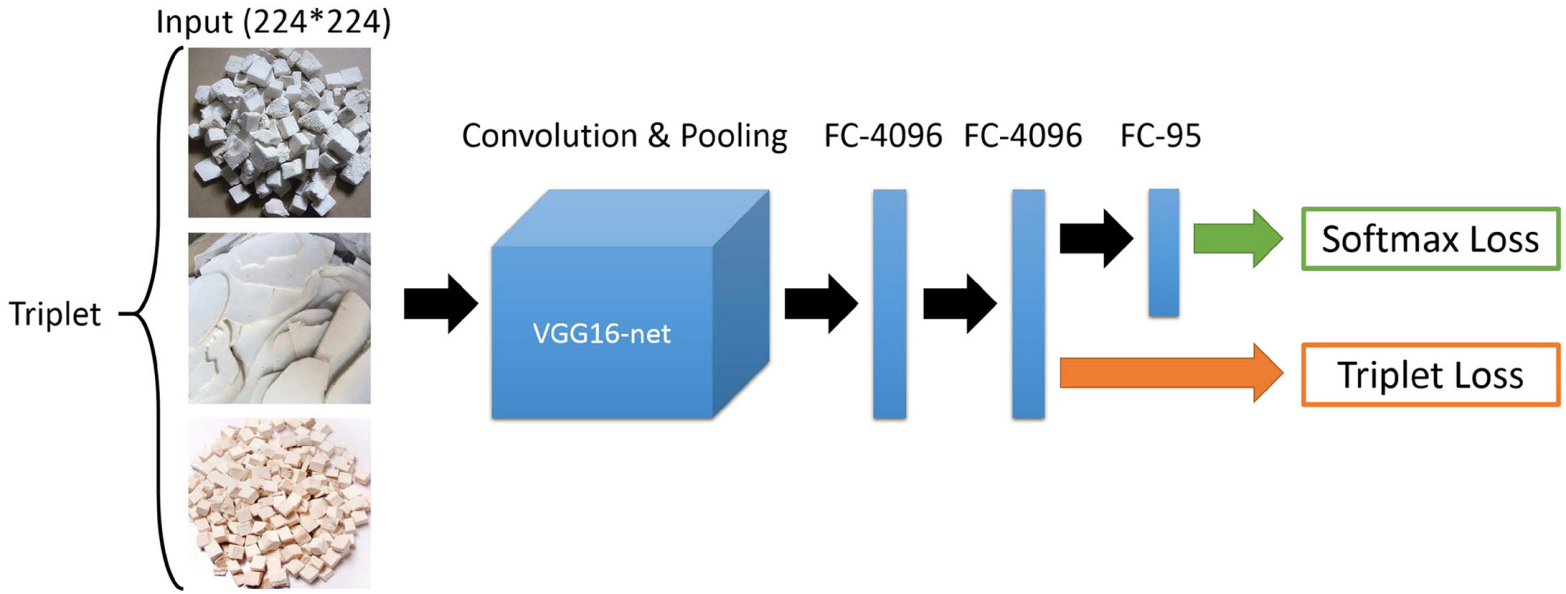
An illustration of training the retrieval CNN model by adding a triplet loss in the pre-trained recognition model.

One way to satisfy this is to train a retrieval network by adding a retrieval loss in the recognition framework, which is actually fine-tuning the recognition network with the retrieval loss, as shown in Fig 5. The typical retrieval loss is the triplet loss, which makes similarity large between similar images and small between dissimilar images. In this way, our model can optimize the softmax loss and retrieval loss jointly, which can search for the appearance similar images under the condition that the images belong to the same medicine category.

Before giving the definition of the triplet loss, we first define the similarity of two images *P* and *Q* according to their squared Euclidean distance:

$$\begin{array}{c} D\left(f\left(P\right),f\left(Q\right)\right)={\left\|f(P)-f(Q)\right\|}_{2}^{2}, \end{array}$$ (1)

where *f*(.) is the function that maps an image to the image representation in the Euclidean space, and *D*(.,.) is the squared Euclidean distance between two images. The smaller the distance *D*(*P*, *Q*) is, the more similar the two images *P* and *Q* are. In the triplet loss, our goal is to learn a mapping function *f*(.) that assigns smaller distance to more similar image pairs, which can be expressed as:

$$\begin{array}{c} D\left(f\left(p_{i}\right),f\left(p_{i}^{+}\right)\right)<D\left(f\left(p_{i}\right),f\left(p_{i}^{-}\right)\right) \end{array}$$ (2)

We call $t_{i}=\left(p_{i},p_{i}^{+},p_{i}^{-}\right)$ a triplet, where *p*_*i*_, $p_{i}^{+}$ and $p_{i}^{-}$ are the query image, similar image and disimilar image respectively. A triplet characterizes a relative similarity ranking order for the images *p*_*i*_, $p_{i}^{+}$ and $p_{i}^{-}$. We can define the triplet loss as:

$$\begin{array}{c} \begin{array}{c} l(p_{i},p_{i}^{+},p_{i}^{-})=\\ \max\left\{0,g+D\left(f\left(p_{i}\right),f\left(p_{i}^{+}\right)\right)-D\left(f\left(p_{i}\right),f\left(p_{i}^{-}\right)\right)\right\} \end{array} \end{array}$$ (3)

where *g* is a gap parameter that regularizes the gap between the distance of the two image pairs: $\left(p_{i},p_{i}^{+}\right)$ and $\left(p_{i},p_{i}^{-}\right)$. The triplet loss is actually a hinge loss, which is a convex approximation to the 0–1 ranking error loss and measures the model’s violation of the ranking order specified in the triplet. In this model, the most crucial component is to learn the mapping function *f*(.). Traditional methods typically employ hand-crafted image features, and learn linear or nonlinear transformations to obtain the mapping function. In this paper, inspired by the robust image representation of CNN, we combine the triplet loss with CNN to learn image similarity models directly from images.

## Experimental Evaluation

In this section, we give the experimental evaluation of our method. We first introduce the detailed experimental settings, then the main results of herbal medicine image recognition and retrieval are given.

### Detailed Settings

**Database**: Our database consists of 95 Chinese medicine categories with 5523 real world herbal medicine images, which are all acquired from the Internet. Most our images contain multiple pieces of mutually occluded herbal and cluttered backgrounds, which make it rather difficult for recognition. For each category, the number of herbal images varies from 30 to 90, and we use all the categories in training and evaluation. In the training phase, we split the database into training and testing set, and we randomly select the training samples by 10 times for training the CNN models; while in the testing phase, the test precision is given by averaging the results on the corresponding 10 testing sets.

**Image Preprocessing**: To train the CNN model, we use color images as input. The reason is that different from previous studies considering handly designed features (color [5], shape [5] and texture [5, 11]), CNN can automatically learn highly semantic information by its deep hierarchical structure, *i.e.*, CNN will learn what is the most important to distinguish different medicine categories. Considering the fact that color is visually important to recognize a medicine category, we use color images to make CNN itself to decide whether or not to learn useful information from color.

For each input image, we first down-sample the images to a fixed resolution of 256 ∗ 256, which is 32 pixels larger than the input image size of the VGG16-net. Then, as the usual settings in training CNN [29, 29, 30], we subtract the image with the mean value of each RGB channel, which will make the CNN model invariant to illumination and easier to converge. Finally, we randomly crop the 224 ∗ 224 patch from the 256 ∗ 256 image to train the CNN model. Particularly, the crop operation is important because it can largely increase the number of training samples, which can improve the generalization of the CNN model under various testing conditions.

**Recognition**: The CNN model is trained using Stochastic Gradient Descent (SGD) [47] with a batch size of 128 examples, momentum of 0.9, and weight decay of 0.0005. This small amount of weight decay is important for the model to learn. We initialized the weights in the last fully-connected layer from a zero-mean Gaussian distribution with the standard deviation of 0.01. For the layers before *conv*3_1, we do not learn them by setting their learning rates to be 0, because these layers contain the important low-level information, which can be commonly used in many object categories. For the other layers, we use an equal learning rate, which we adjust manually throughout training. The heuristic we follow is to divide the learning rate by 10 when the error rate stopped improving with the current learning rate. The starting learning rate of the VGG16-net is 0.01, thus our starting rate should be lower, which is set to be 0.001 and reduced two times prior to the termination. We train the network for 40 epochs throughout the training set, which takes about three hours on a single NVIDIA Titan X GPU.

**Retrieval**: The same to the training of the recognition network, we use the same momentum and weight decay in training the retrieval network, and we do not train the layers before *conv*3_1 for the same reason. Due to the use of triplet loss in training, we reduce the batchsize in training the recognition network by 3, *i.e.*, we use the batchsize of 40 to avoid large GPU memory cost. Besides, the margin *g* is important to tune the retrieval model, and we set the margin to be 5 in the triplet loss. To train the network, we set the starting learning rate to be 0.0001, which divides the starting learning rate of the recognition network by 10 to preserve the recognition ability. Finally, we train the network for 40 epochs throughout the training set, which takes about three hours on a single NVIDIA Titan X GPU.

**Compared Methods**: For comparison, we compare our method with three main previous studies, which use shape, texture and color for herbal medicine image recognition. For shape and texture, Li [5] used the Bag-of-Words model with the local shape and texture features to generate image representation. As the same in their setting, we adopt Scale Invariant Feature Transformation (SIFT) [12] and Local Binary Patterns (LBP) [15] descriptors to describe the shape and texture respectively. Besides, considering the much more medicine categories we have, we use 100 clusters in the vocabulary construction to cover all the categories, then we use the typical hard voting and average pooling to generate the final image presentation. For color, we transfer the original RGB image into the HSV color space as used in [5], and the color space is further divided into multiple subspaces. In our implementation, we use 8 subspaces for each HSV channel, and the image representation is generated by summing over the pixels located in each subspace.

**Evaluation**: For the recognition problem, we use the classification precision in the test set for evaluation. The precision is computed by dividing the total number of test samples with the number of correctly classified test samples. We report the classification precision of each medicine category and average classification precision of all the 95 categories. For the retrieval problem, we also use the classification precision, while the difference is that we compute classification precision on the top ranking images of query images.

### Recognition Results

**Fig 6:**
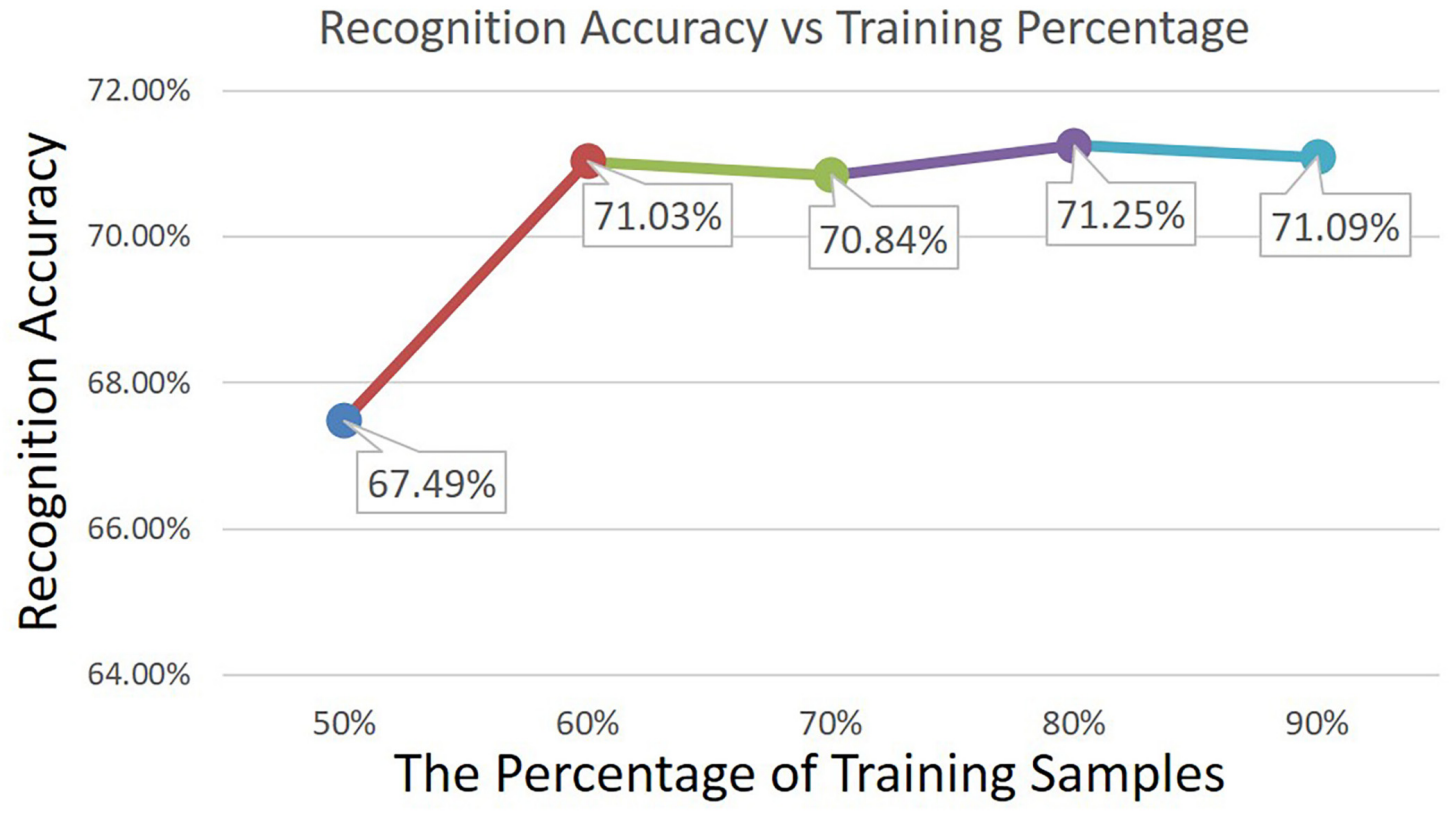
The average recognition precision of all the 95 medicine categories base on the different percentage of training samples (50%∼90%).

In this part, we give the experimental results on herbal medicine image recognition. Fig 6 shows the average recognition precision of all the 95 medicine categories in the test set, and the percentage of training samples varies from 50% to 90%. It can be observed that with only 50% training images, our method can achieve the average precision of 67.49%, and this result is promising given the fact that most images have multiple pieces of mutually occluded herbal. It also indicates that our method is able to learn the difference of many medicine categories with only a small number of training images. As the training images becomes more, the precision gets a large improvement and remains stable, *e.g.*, the precision is about 71.03% with 60% training images and 71.09% with 90% training images. This result provides a application guideline for the recognition problem, in which labeling all the training images is not necessary while labeling 60% samples is already enough, and this will save a lot of human efforts and money in labeling medicine images.

**Fig 7:**
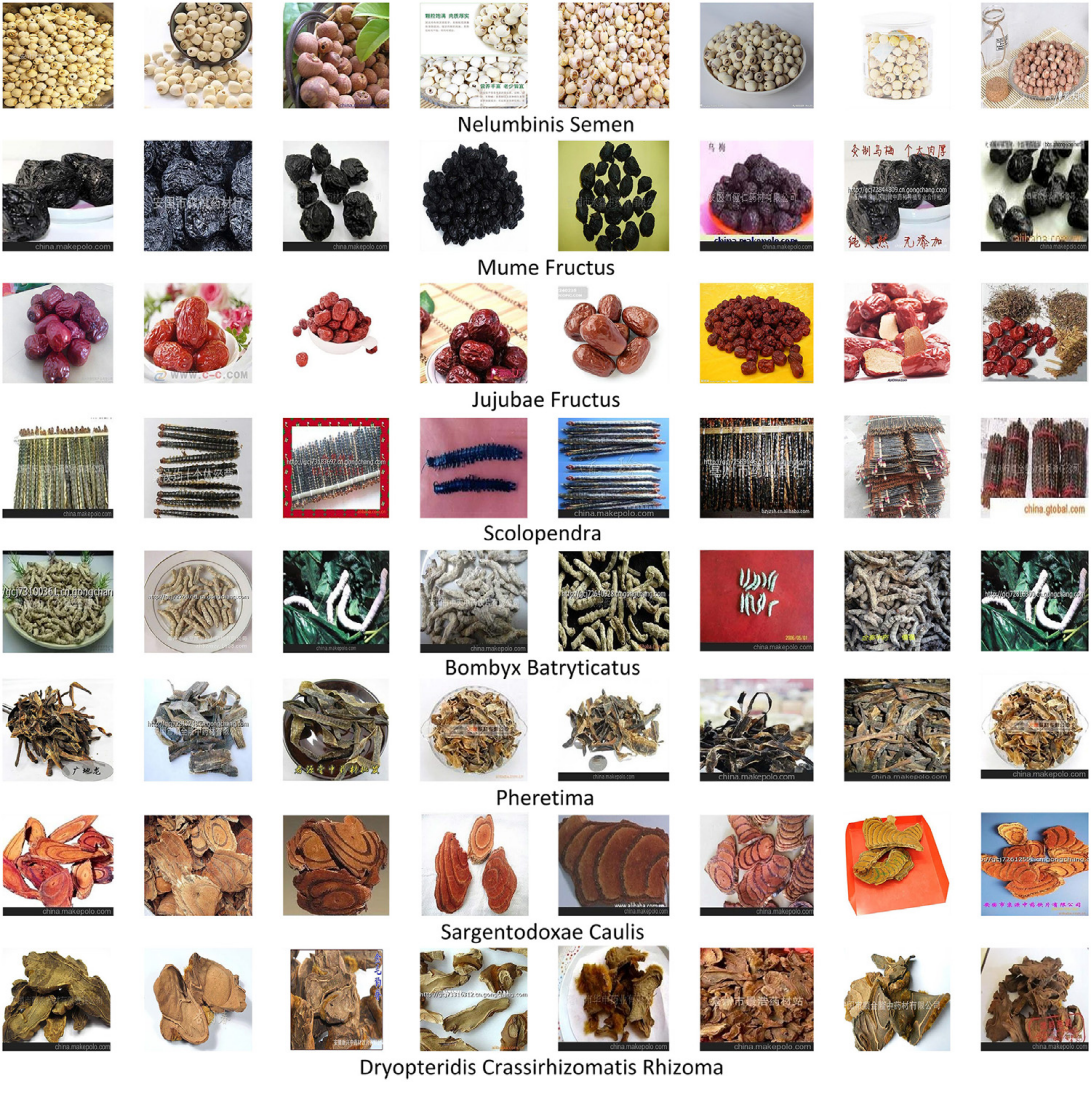
Some correctly recognized medicine images by the recognition CNN model trained with 60% training images. Each row represents the herbal images predicted to be the same category.

Fig 7 shows some examples of the correctly recognized herbal medicine images, and each row represents the images with the same category. It can be observed that although these images have multiple pieces of mutually occluded herbal and cluttered backgrounds, our method can overcome these challenges and give promising recognition, which demonstrates that the Convolutional Neural Network (CNN) is able to construct much more robust image representation than the low-level ones in previous studies. To understand the recognition difficulty of each medicine category, we also give the recognition precision for each category on the test set under 60% training samples, as shown in Table 2. The numbers behind the precision in the bracket indicate the number of correctly recognized herbal medicine images in the total number of test images for this category. It can be observed some medicine categories get very high recognition precision, *e.g.*, Chrysanthemi Flos, Carthami Flos, Scolopendra and Nelumbinis Plumula get 100% precision; while the precision is relatively low for some categories, *e.g.*, Arctii Fructus and Fraxini Cortex only obtain the precision around 20%.

**Table 2:** The recognition precision of each Chinese herbal medicine category with 60% training samples. The numbers in the bracket indicate the number of correctly recognized images in the total number of test images for each category.

Cinnamomi Ramulus	Zingiberis Rhizoma Recens	Xanthii Fructus	Magnoliae Flos	Asari Radix Et Rhizoma
70.73% (29/41)	97.22% (35/36)	53.84% (7/13)	83.33% (25/30)	45.45% (10/22)
Menthae Haplocalycis Herba	Arctii Fructus	Mori Folium	Chrysanthemi Flos	Cicadae Periostracum
23.80% (5/21)	22.22% (2/9)	88.89% (8/9)	100% (21/21)	92.59% (25/27)
Puerariae Thomsonii Radix	Bupleuri Radix	Cimicifugae Rhizoma	Spirodelae Herba	Gypsum Fibrosum
45.83% (11/14)	52.38% (11/21)	61.53% (8/13)	66.67% (6/9)	55.56% (5/9)
Anemarrhenae Rhizoma	Phragmitis Rhizoma	Trichosanthis Radix	Gardeniae Fructus	Cassiae Semen
41.17% (7/17)	85.71% (24/28)	76.67% (23/30)	90.47% (19/21)	50% (7/14)
Coptidis Rhizoma	Phellodendri Chinensis Cortex	Gentianae Radix Et Rhizoma	Fraxini Cortex	Lonicerae Japonicae Flos
70.83% (17/24)	78.57% (22/28)	41.67% (5/12)	21.42% (3/14)	93.33% (28/30)
Forsythiae Fructus	Isatidis Radix	Dryopteridis Crassirhizomatis Rhizoma	Rhei Radix Et Rhizoma	Sargentodoxae Caulis
88.89% (16/18)	52.17% (12/23)	50% (6/12)	77.14% (27/35)	96.67% (29/30)
Rehmanniae Radix	Moutan Cortex	Artemisiae Annuae Herba	Cynanchi Atrati Radix Et Rhizoma	Natrii Sulfas
57.14% (12/21)	58.82% (10/17)	35.71% (5/14)	78.26% (18/23)	50% (4/8)
Aloe	Chaenomelis Fructus	Zaocys	Cibotii Rhizoma	Magnoliae Officinalis Cortex
33.33% (2/6)	58.82% (10/17)	88.89% (24/27)	92.68% (38/41)	85.71% (12/14)
Tsaoko Fructus	Poria	Artemisiae Scopariae Herba	Aconiti Lateralis Radix Praeparata	Cinnamomi Cortex
90.62% (29/32)	77.14% (27/35)	64% (16/25)	40% (8/20)	56.25% (9/16)
Caryophylli Flos	Citri Reticulatae Pericarpium	Aurantii Fructus Immaturus	Citri Sarcodactylis Fructus	Toosendan Fructus
50% (6/12)	62.96% (17/27)	78.78% (26/33)	58.82% (10/17)	81.81% (18/22)
Crataegi Fructus	Hordei Fructus Germinatus	Quisqualis Fructus	Arecae Semen	Semen Cucurbitae
73.68% (14/19)	80% (8/10)	68.75% (11/16)	83.33% (10/12)	60% (6/10)
sophorae Flos	Artemisiae Argyi Folium	Chuanxiong Rhizoma	Curcumae Radix	Olibanum
33.33% (3/9)	76.67% (23/30)	42.46% (31/73)	75% (15/20)	70.83% (17/24)
Carthami Flos	Leonuri Herba	Spatholobi Caulis	Pinelliae Rhizoma	Uncariae Ramulus Cum Uncis
100% (14/14)	87.5% (7/8)	96.87% (31/32)	51.85% (14/27)	89.28% (25/28)
Fritillariae Cirrhosae Bulbus	Ginkgo Semen	Tribuli Fructus	Ziziphi Spinosae Semen	Ostreae Concha
45% (18/40)	86.67% (26/30)	63.15% (12/19)	65% (13/20)	72.72% (16/22)
Apocyni Veneti Folium	Pheretima	Gastrodiae Rhizoma	Bombyx Batryticatus	Scorpio
60.86% (14/23)	76.59% (36/47)	58.62% (17/29)	76.31% (29/38)	80.55% (29/36)
Scolopendra	Acori Tatarinowii Rhizoma	borneolum Syntheticum	Ginseng Radix Et Rhizoma	Panacis Quinquefolii Radix
100% (43/43)	53.12% (17/32)	57.14% (8/14)	75% (15/20)	69.23% (18/26)
Astragali Radix	Atractylodis Macrocephalae Rhizoma	Gecko	Rehmanniae Radix Praeparata	Angelicae Sinensis Radix
70% (21/30)	62.5% (15/24)	72.72% (8/11)	10% (1/10)	19.23% (10/52)
Paeoniae Radix Alba	Ophiopogonis Radix	Ganoderma	Jujubae Fructus	Trionycis Carapax
73.07% (19/26)	86.36% (19/22)	76% (19/25)	95.65% (44/46)	83.33% (25/30)
Mume Fructus	Euryales Semen	Nelumbinis Semen	Nelumbinis Plumula	Schisandrae Chinensis Fructus
97.29% (36/37)	83.33% (10/12)	90.91% (40/44)	100% (17/17)	70% (14/20)

We observe that the categories with high precision usually contain the herbal with stable shape and similar appearance, which is easy for training and recognition, while herbal in the categories with low precision varies a lot in size and shape. More importantly, some herbal have totally different appearance, and even the herbal from other categories seems more similar, which makes the CNN very difficult to train. Actually, in our first submission, most herbals in Zingiberis Rhizoma Recens are slices and whole pieces, and considering the fact that we have only a few images for training, it is hard for CNN to learn the difference with such few samples (15 for training) and the recognition precision is only about 10%. However, after we removed most of the slices and largely increase the number of training samples for the whole pieces, the precision has been significantly improved to 97.22%, which demonstrates that CNN can work well with more training samples and less inner-class diversity. Therefore, inner-class diversity is an important factor for medicine herbal recognition as we cannot consider only one specific shape of the herbal in practical applications, and we will try our best to address this problem in our future study.

The number of training iterations is also important for recognition. In our experiments, we use two learning rates in training (0.001 and 0.0001), in which 3000 iterations are used for each learning rate, and there are in total 6000 iterations to train the CNN recognition model. Table 3 shows the average classification precision with different iterations for different number of training samples. We can see clearly that no matter how the number of training samples changes, the decrease of learning rate will result in an obvious improvement in the recognition precision. Besides, the highest precision always happen under the lower learning rate of 0.0001, *e.g.*, 5000 iterations are the best for 50% and 60% training images, while 6000 iterations are appropriate for 70%∼90% training samples. These results imply that the starting learning rate is usually used to learn a rough model, while the lower learning rate is used to refine the model to the local minima. Therefore, it would be better to use two or three learning rates to learn the recognition model, and due to the limited of training images, two learning rates are enough to give reasonable results and avoid over-fitting in training.

**Table 3:** The average recognition precision of all the 95 medicine categories on the test set. The precision is given under different percentage of training samples, and each percentage is evaluated with different training iterations.

	1000	2000	3000	4000	5000	6000
**50%**	65.27%	65.59%	65.52%	67.12%	***67.49%***	67.41%
**60%**	66.79%	68.19%	68.73%	70.31%	***71.13%***	70.85%
**70%**	66.48%	69.52%	69.34%	70.41%	70.53%	***70.84%***
**80%**	67.98%	70.19%	69.22%	71.08%	71.25%	***71.25%***
**90%**	67.84%	68.18%	68.18%	71.16%	71.06%	***71.06%***

**Fig 8:**
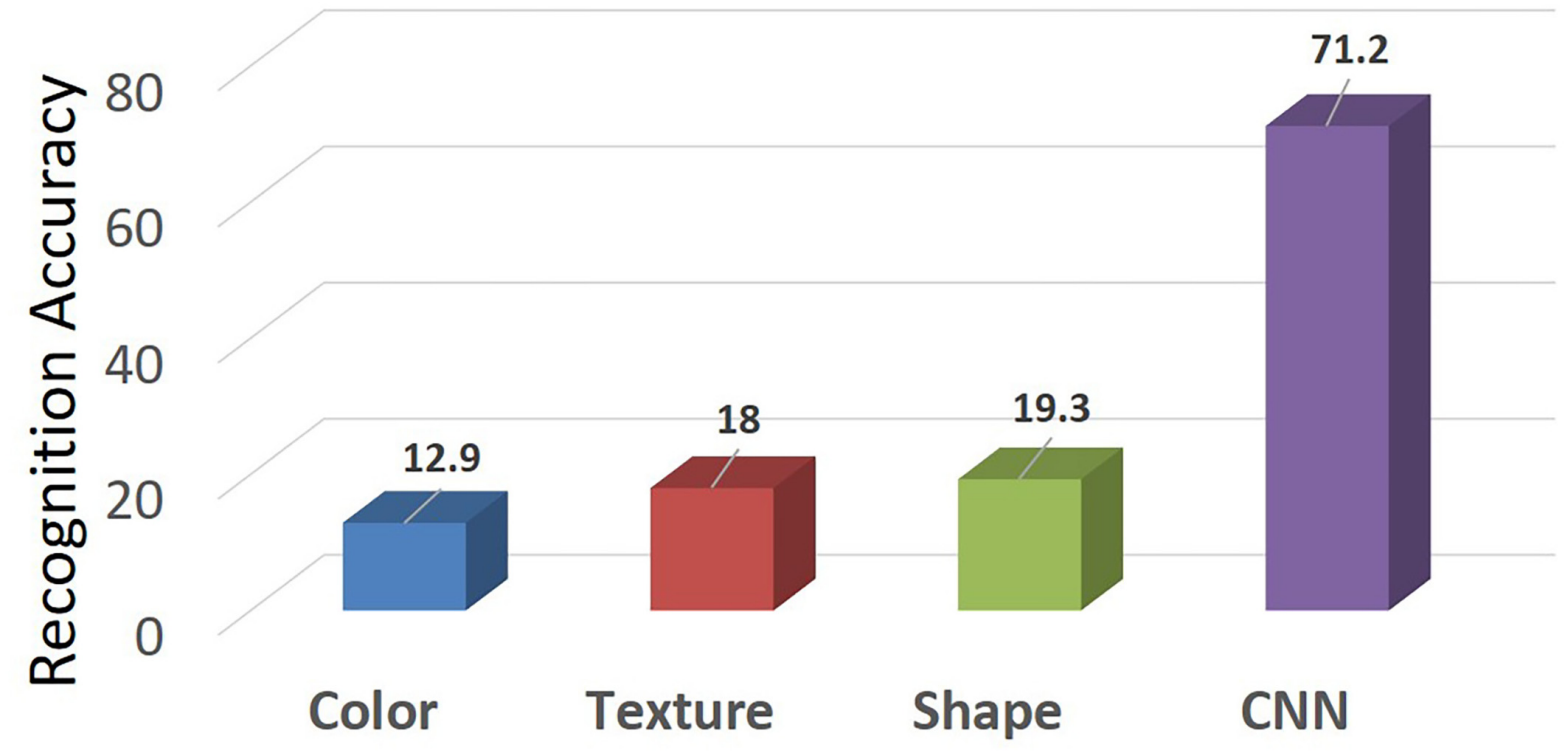
The comparison of recognition precision between our proposed method and three main previous studies. The recognition precision is given under the same experimental setting to the above ones in which 60% samples are used for training.

To see how much we have improved over other methods, Fig 8 shows the comparison between our method (CNN) and three main previous studies, which use hand-crafted color, texture and shape features respectively. It can be clearly observed that our method has improved the recognition precision by a large margin, *i.e.*, from 19.3% to 71.2%. The reason for this larger improvement is that the previous studies only consider handly designed low-level (SIFT, LBP [12, 15]) and mid-level image representation (Bag-of-Words model [5, 18–21]), while these representation cannot distinguish a large number of medicine categories, *e.g.*, they consider 18 categories at most. However, the CNN based image representation can learn high level semantic of these categories by deep hierarchical structures, which are more robust to object variations such as illumination, multiple pieces of occluded herbal and cluttered backgrounds.

### Retrieval Results

**Fig 9:**
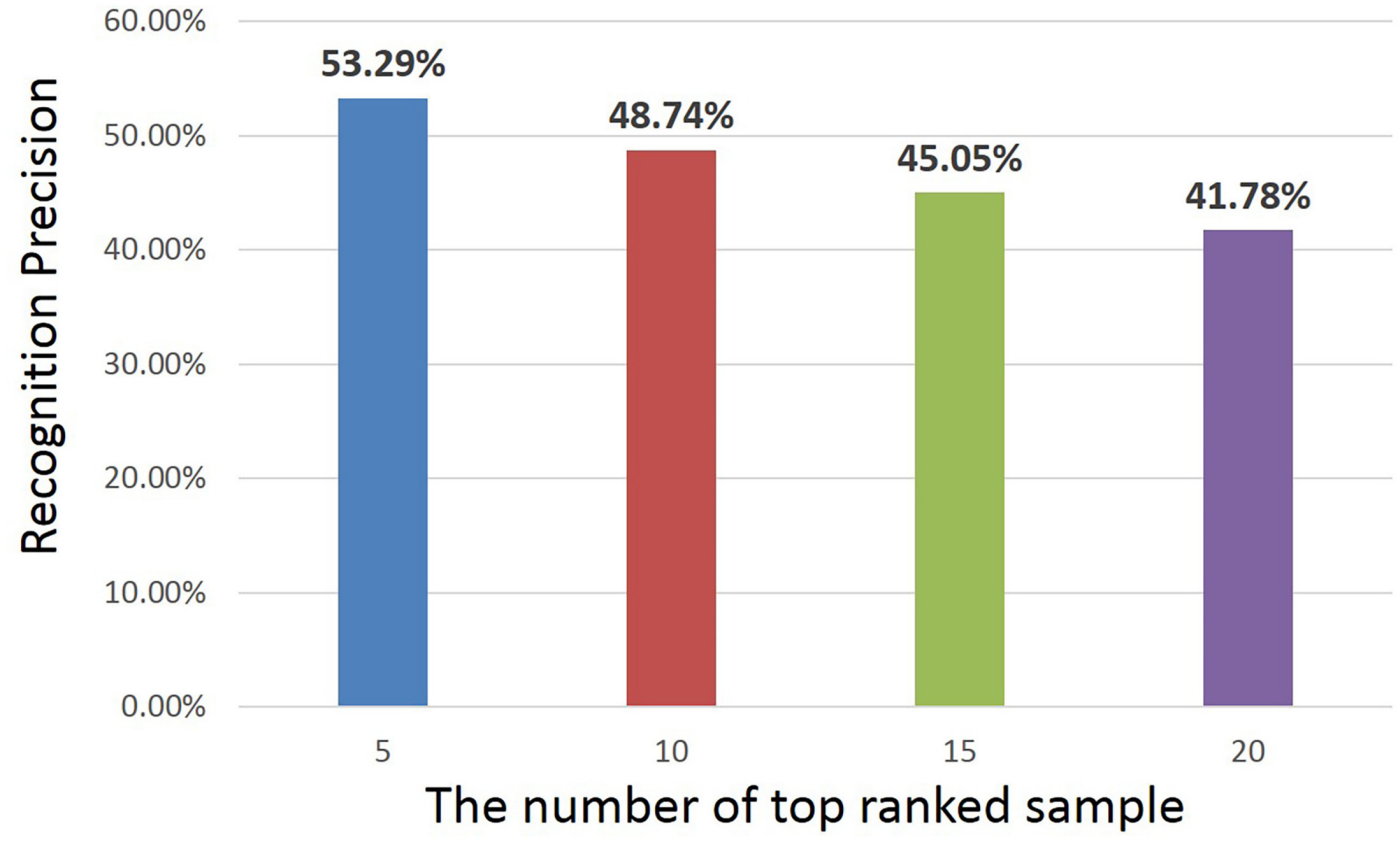
The retrieval precision of all the 95 medicine categories on the test set. The precision is given under 60% training images with different number of top ranking images (5 ∼ 20).

In this part, we give the detailed experimental results on herbal medicine image retrieval. Fig 9 shows the retrieval precision of all the 95 medicine categories on the test set, and the precision is given with different number of top ranking images (5 ∼ 20) under 60% training images. There are two reasons we only use 60% training samples for retrieval. Firstly, we have to guarantee the searching set is large enough, and the searching set is the test set. If we use 90% training images, there will be only 10% for test, and some categories may not have more than 5 test images, which cannot be evaluated in retrieval. Secondly, based on the recognition precision in Fig 6, 60% training images are already enough to give promising recognition performance, thus it is not necessary to use more training data. It can be observed from Fig 9 that as the number of top ranking images becomes larger, the retrieval precision keeps decreasing, *e.g.*, the precision of the top 5 is 53.29%, while it decreases to 41.78% when using top 20. This is because the top ranking images are the confident ones given by the retrieval model, but as more images are searched, more images that are not the correct ones will be included.

**Fig 10:**
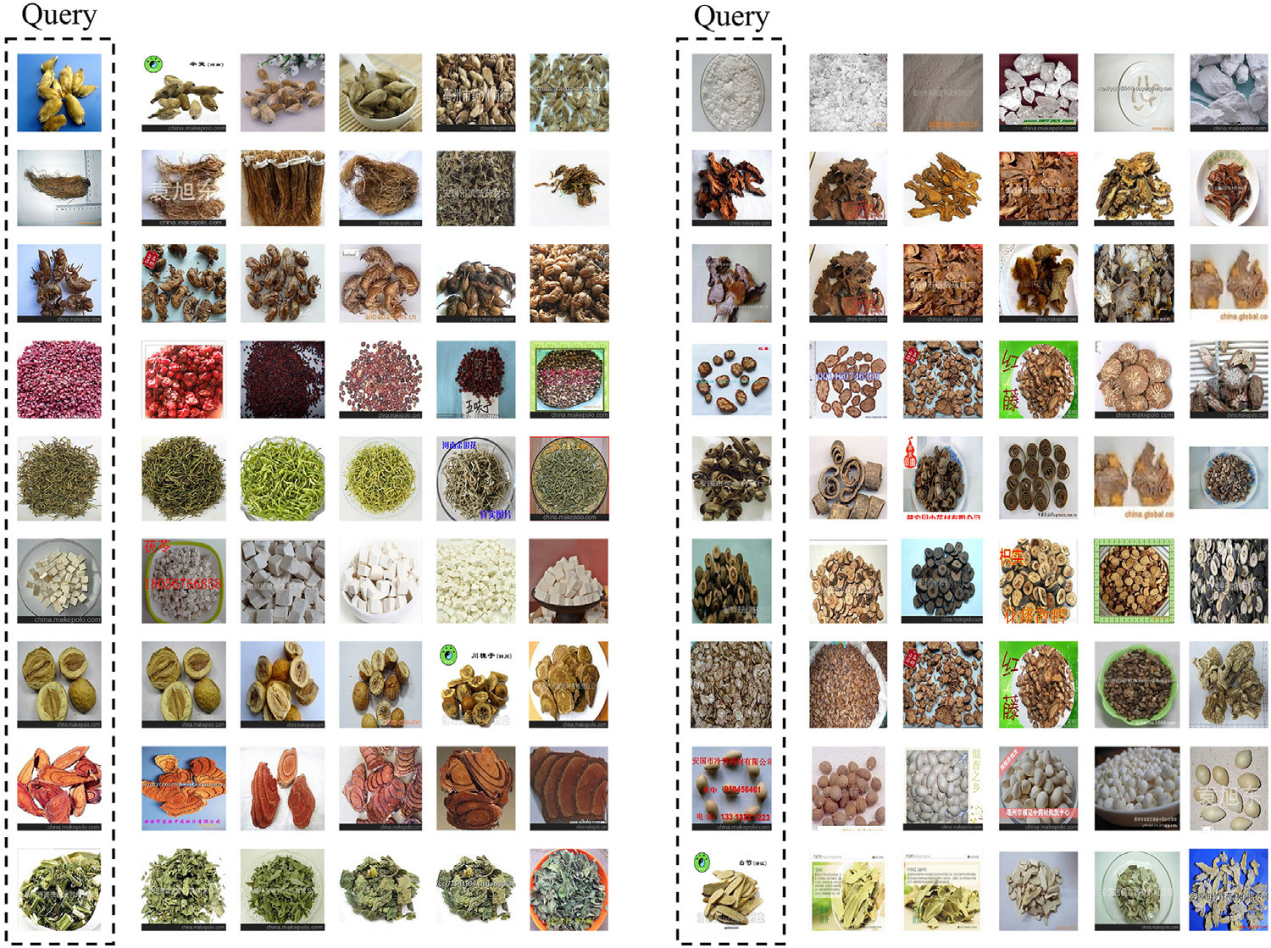
Some retrieval results of the Chinese herbal medicine images by the retrieval CNN model trained with 60% training images. The first image is the query image, and the top 5 ranking images of each query image are shown.

To see the influence of different iterations in training, Table 4 lists the retrieval precision with different iterations and different number of top ranking images under 60% training images. Similar to the experiments in recognition, we also use two learning rates but lower than the ones in recognition, *i.e.*, 0.0001 and 0.00001 are used. Each learning rate has 3000 iterations, and there are in total 6000 iterations to train the retrieval model. It can be observed that the best retrieval performance is always obtained under 6000 iterations, no matter how the number of top ranking images changes. The reason for this is that training the retrieval model has to optimize two optimization objectives jointly, *i.e.*, the softmax loss and triplet loss, which will take a longer time to converge. Finally, Fig 10 shows some retrieval results with the top 5 ranking images under 60% training images. We can see that for most herbal medicine images, we can give promising retrieval results although the herbal are mutually occluded and vary a lot in shape and size.

**Table 4:** The retrieval precision under 60% training images with different number of top ranking images (5 ∼ 20) and different number of training iterations (1000 ∼ 6000).

	1000	2000	3000	4000	5000	6000
**top 5**	51.85%	52.08%	53.09%	53.28%	53.13%	***53.29%***
**top 10**	46.68%	47.10%	48.44%	48.69%	48.62%	***48.74%***
**top 15**	43.10%	43.40%	44.65%	44.87%	44.77%	***45.05%***
**top 20**	39.57%	40.01%	41.25%	41.65%	41.74%	***41.78%***

**Fig 11:**
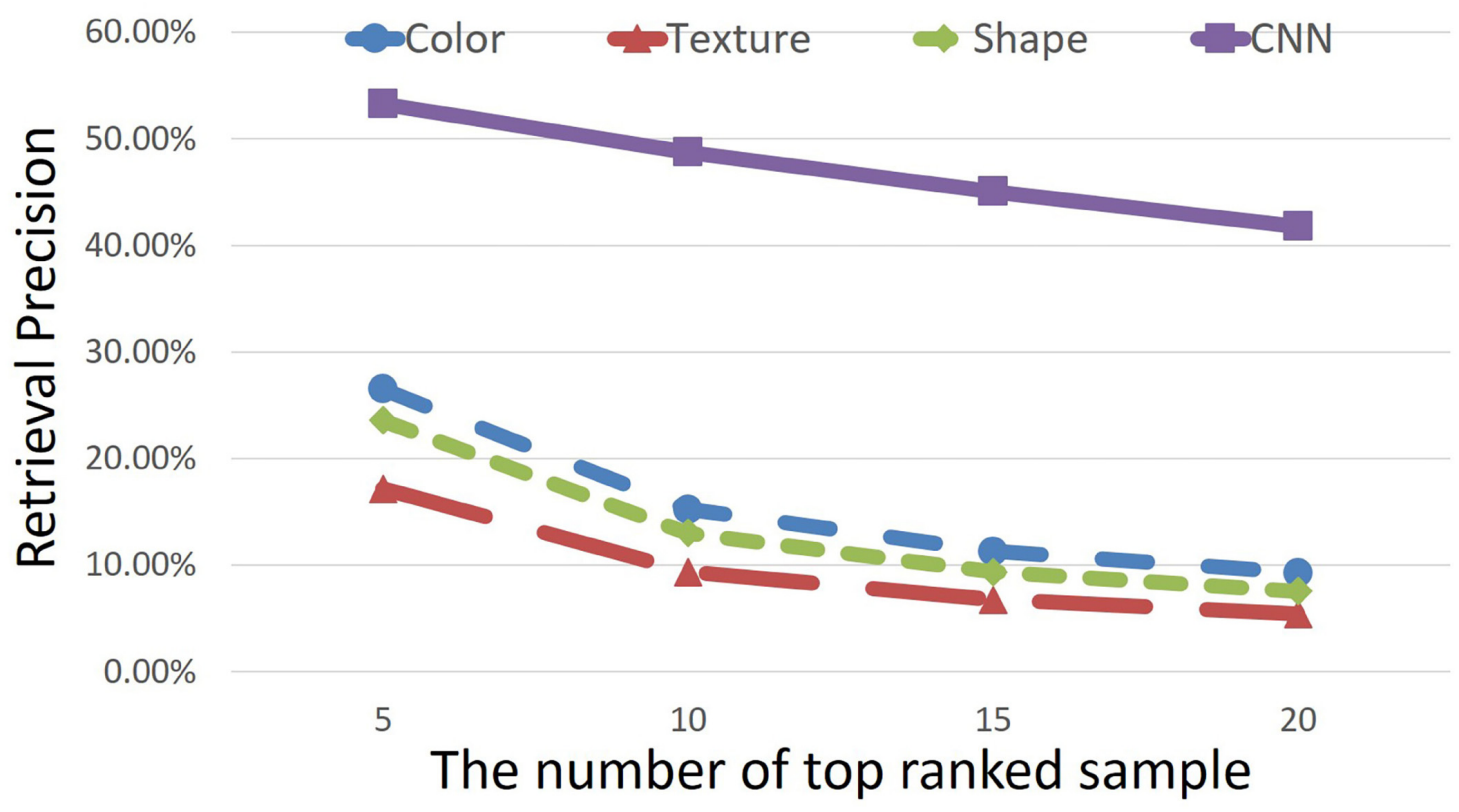
The comparison of retrieval precision between our proposed method and three main previous studies. The retrieval precision is given under the same experimental setting to the above ones in which 60% samples are used for training.

To see the comparison with previous methods, Fig 11 shows the comparison between our method and other three main previous studies. For detailed value, please refer to S1 Table. Due to the little study in Chinese herbal image retrieval, we use the same three previous methods as used in the comparison of the recognition task. Although these studies are not designed for the retrieval task, the evaluation still can give some useful information about how powerful the convolutional neural network can be. We see clearly that our method again obtains a very large lead, which improves the methods of “color” by doubling its precision. As the number of top samples increases, our improvement becomes much larger, which implies that the low-level and mid-level features are easily affected by the inter-class variations brought by the herbal of other categories, while CNN can give relatively stable and robust prediction.

## Conclusion

In this paper, we have studied two practical problems on Chinese medicine: Chinese herbal medicine image recognition and retrieval. We have proposed to use the Convolutional Neural Network (CNN) in the machine learning and computer vision community to solve the above problems. Firstly, we have constructed a public herbal image database with multiple pieces of mutually occluded herbal and cluttered backgrounds. Compared with the previous studies which only consider single clean herbal, our database has provided the same standard for evaluating different methods and pushed the methods into practical applications. Then, for the recognition problem, we have used the softmax loss to optimize the recognition CNN model, which can generate much more robust image representation than low-level features. Finally, for the retrieval problem, we have proposed to fine-tune the above recognition network by adding a triplet loss for training the retrieval network, which has considered both semantic and appearance information to give accurate search. Experimental results have shown that our method can achieve the average recognition precision of 71% and the average retrieval precision of 53% over all the 95 medicine categories, which are quite promising given the fact the images have multiple pieces of mutually occluded herbal and cluttered backgrounds. In our future work, we will consider the inner-class diversity to further enhance recognition and retrieval.

## Supporting Information

S1 Table The corresponding value of data for Fig 11. (PDF)
