# Supplementary material for: Chinese Herbal Medicine Image Recognition and Retrieval by Convolutional Neural Network
Source: PLoS One. 2016 Jun 3;11(6):e0156327. doi: 10.1371/journal.pone.0156327 (PMC4892594; doi:10.1371/journal.pone.0156327)
Supplement: S1 Table — (PDF) [file pone.0156327.s001.pdf]

**Table 1.** The corresponding value of data for Fig.11.

| | top 5 | top 10 | top 15 | top 20 |
|---|---|---|---|---|
| CNN | 53.29% | 48.74% | 45.05% | 41.78% |
| Color | 26.52% | 15.24% | 11.29% | 9.27% |
| Texture | 17.17% | 9.37% | 6.75% | 5.41% |
| Shape | 23.59% | 13.02% | 9.38% | 7.55% |
